# Heme oxygenase-1 attenuates cadmium-induced mitochondrial-caspase 3- dependent apoptosis in human hepatoma cell line

**DOI:** 10.1186/s40360-015-0040-y

**Published:** 2015-12-15

**Authors:** Akeem O. Lawal, Jeanine L. Marnewick, Elizabeth M. Ellis

**Affiliations:** Oxidative Stress Research Centre, Faculty of Health and Wellness Sciences, Cape Peninsula University of Technology, Bellville Campus, Bellville, 7535 South Africa; Institute of Biomedical and Microbial Biotechnology, Cape Peninsula University of Technology, Bellville Campus, Bellville, 7535 South Africa; Strathclyde Institute of Pharmacy and Biomedical Sciences, University of Strathclyde, G1 1XW Glasgow, UK

**Keywords:** Cadmium, Heme oxygenase-1, Caspase-3, Cytochrome c, Apoptosis, Human hepatoma cells

## Abstract

**Background:**

Cadmium (Cd) is a well known environmental and industrial toxicant causing damaging effects in numerous organs. In this study, we examined the role of heme oxygenase-1 (HO-1) in modulating the Cd-induced apoptosis in human hepatoma (HepG2) cells after 24 h exposure.

**Methods:**

HepG2 cells were exposed to 5 and 10 μM Cd as CdCl_2_ for 24 h while other sets of cells were pre-treated with either 10 μM Cobalt protoporphyrin (CoPPIX) or 10 μM Tin protoporphyrin (SnPPIX) for 24 h, or 50 μM Z-DEVD-FMK for 1 h before exposure to 5 and 10 μM CdCl_2_ for 24 h. Expressions of caspase 3, cytosolic cytochrome c, mitochondrial Bax and anti-apoptotic BCL-xl proteins were assessed by western blot. Intracellular reactive oxygen species (ROS) production was determined using the dihydrofluorescein diacetate (H_2_DFA) method. Cell viability was assessed by MTT assay, while a flow cytometry method was used to assess the level of apoptosis in the cell populations.

**Results:**

Our results show that there were a significant increase in the expression of cytosolic cytochrome c, mitochondrial Bax protein, and caspase 3 at 5 and 10 μM compared to the control, but these increases were attenuated by the presence of CoPPIX. The presence of SnPPIX significantly enhanced Cd-induced caspase 3 activities. CoPPIX significantly decreased the level of ROS production by 24.6 and 22.2 % in 5 and 10 μM CdCl_2,_ respectively, but SnPPIX caused a significant increase in ROS production in the presence of CdCl_2_. HepG2 cell viability was also significantly impaired by 13.89 and 32.53 % in the presence of 5 and 10 μM CdCl_2_, respectively, but the presence of CoPPIX and Z-DEVD-FMK significantly enhanced cell survival, while SnPPIX enhanced Cd-impaired cell viability. The presence of CoPPIX and Z-DEVD-FMK also significantly decreased the population of apoptotic and necrotic cells compared with Cd.

**Conclusion:**

In summary, the present study showed that HO-1 attenuates the Cd-induced caspase 3 dependent pathway of apoptosis in HepG2 cells, probably by modulating Cd-induced oxidative stress.

## Background

Cadmium (Cd) has been referred to as a group 1 carcinogen and a potential human carcinogen with an estimated half-life of 15 to 20 years [1, 2]. This implies that the metal escapes detoxification processes and therefore makes it a more potent toxicant in the body. Cd is a nonessential heavy metal found in the earth’s crust in association with zinc ores. It is an environmental and industrial toxicant inducing multi-organ damaging effects. It is not naturally abundant and does not degrade in the environment causing a constant increasing risk of human exposure [3–5]. The toxicity of Cd has been well established in several *in vivo* and *in vitro* studies [6–12]. Though the toxicity of this heavy metal is well established, its mechanism of action is not fully elucidated. Different studies have implicated the involvement of either caspase-dependent and -independent or both pathways in Cd toxicity, depending on the study models, period and dose of exposure [10, 13–16]. In addition, studies have implicated the generation of reactive oxygen species (ROS) as an important mechanism in Cd-induced toxicity [17–19].

Heavy metals, including Cd, exert their toxicity by targeting mitochondria [20]. Several studies have shown that Cd exposure triggers the caspase-dependent pathway causing elevated levels of cytosolic cytochrome c, mitochondrial Bax protein and caspase 9 activation with consequent activation of executioner caspase 3 [13–15, 21, 22]. Cd telluride quantum dots (CdTe-QDs) have been shown to induce apoptosis, with elevated caspase 3 activity, decreased Bcl-2, increased cytosolic cytochrome c and increased mitochondrial Bax protein level, in HepG2 cells [23]. In the caspase-independent pathway, Cd binds to the thiol groups of proteins in the mitochondrial membrane, thereby affecting mitochondrial membrane permeability with a resultant increase in ROS generation [24, 25]. The ROS can trigger mitochondrial permeability transition resulting in apoptosis and necrosis. The majority of intracellular ROS produced is from mitochondrial respiration and results from the disturbance of the mitochondrial electron transport chain by chemicals such as Cd. The disturbance to the mitochondrial membrane results in the leakage of electrons to the molecular oxygen to produce ROS (such as superoxide anion).

The generation of ROS and development of oxidative stress have been implicated in apoptosis [26]. We have previously shown that Cd triggered a significant increase in ROS production in HepG2 cells [17] and studies have shown the involvement of ROS in caspase activation and apoptosis in different cell lines [26–29]. It is therefore possible that Cd-induced ROS production in HepG2 cells may be responsible for a significant portion of the apoptotic and necrotic effects in human hepatoma cells.

In order to maintain the intracellular redox homeostasis, cells are equipped with antioxidant defense mechanisms that are induced in the presence of excess ROS. One such mechanism is the antioxidant enzyme heme oxygenase-1 (HO-1), an inducible form of heme oxygenase, which catalyses the rate-limiting step catabolism of heme to produce ferrous iron (Fe^2+^), carbon monoxide (CO) and biliverdin [30]. HO-1 has been reported to exert anti-apoptotic, antioxidant, anti-inflammatory and cytoprotective effects in different cell lines [30, 31]. We and others have shown that Cd induced the expression of HO-1 in human astrocytoma 1321 N1 cells [8], mammary epithelial MCF-7 cells [32], and 2937 cells [33], probably in response to oxidative stress induced by the metal. Although it has been shown that HO-1 prevents crotonaldehyde-stimulated apoptosis in HepG2 cells [34], no study has yet shown whether HO-1 can protect HepG2 cells against Cd-induced apoptosis. Also, it was shown that under certain conditions HO-1 overexpression could have a deleterious effect on cells [35] and so it is not yet clear what the role of HO-1 induction in Cd toxicity is. Therefore, in this study, we evaluated the anti-oxidative and anti-apoptotic effects of HO-1 overexpression in human hepatoma cells (HepG2) exposed to the environmental toxic heavy metal, Cd for the purpose of identifying a therapeutic target for dealing with Cd toxicity.

## Materials and methods

### Chemicals and reagents

CdCl_2_ was obtained from Sigma-Aldrich (Poole, Dorset, UK). Antibodies against Tom40, GAPDH, cytochrome c, Bax, BCL-xl, HO-1 and caspase 3 were obtained from Santa Cruz Biotechnology (Middlesex, UK). Horseradish peroxidase-conjugated goat anti-rabbit antibody was obtained from Bio-Rad laboratory (Hempstead, UK). Polyacrylamide (30 %) was purchased from Seven Biotech Ltd (Worcestershire, UK). Nitrocellulose membranes were purchased from Amersham Biosciences (Amersham, Bucks, UK). Caspase 3 and Calpain activity detection kits were obtained from Calbiochem (Nothingham, UK) and Promega (Southampton, UK), respectively. Annexin V-Cy3 Apoptosis detection Kit Plus (Cat # K202-25) was obtained from BioVision (Mountain View, CA, USA). Protoporphrin IX cobalt chloride (CoPPIX), SnPPIX, Z-DEVD-FMK, and all other chemicals were of the highest grade available and were obtained from Sigma-Aldrich (Poole, Dorset, UK).

### Cell culture and treatments

HepG2 human liver hepatoma cells [36] were purchased from the American Type Culture Collection (ATCC, Rockville, MD, USA). The cells were grown in Dulbecco’s modified Eagle’s medium (DMEM) supplemented with 10 % fetal bovine serum (FBS), 1 % MEM nonessential amino acid solution, 1 % sodium pyruvate solution and 1 % penicillin-streptomycin solution. The cells were allowed to grow at 35 °C in a humidified atmosphere of 5 % CO_2_ and 95 % air. Prior to treatment, the cells were replated in an appropriate cell culture plate and allowed to attach for 24 h. Approximately 5 x 10^6^ cells/well (total volume of 2 ml/well) were plated for treatments done in 6 well plates (1 well = 9.6 cm^2^). HepG2 cells were treated with either 5 or 10 μM cadmium as CdCl_2_ (prepared in double distilled water and sterile filtered) for 24 h at 37 °C. After incubation, the cells were harvested for the various assays. For induction and inhibition studies, HepG2 cells were pre-treated with either 10 μM CoPPIX (prepared in 0.1 M NaOH and pH adjusted to 7.4) or 10 μM SnPPIX for 24 h at 37 °C or 50 μM Z-DEVD-FMK (10 mM stock prepared in DMSO and diluted to working solution in culture medium) for 1 h at 37 °C, respectively. After the incubation period, cells were washed with PBS and then exposed to 5 and 10 μM CdCl_2_ for 24 h. The cells were then harvested for the various assays.

### Calpain activity

Calpain activity in whole cell extracts was determined using the Calpain Activity Assay Kit from Calbiochem. The cells were treated with 5 and 10 μM CdCl_2_ for 24 h, and the assay was performed as described in the kit protocol. The assay is based on the release of 7-amino-4-methylcoumarin (AMC) from a synthetic calpain substrate, Suc-Leu-Leu-Val-Tyr-AMC, by calpain. The fluorescence intensity of the cleavage product, AMC, was measured at an excitation wavelength of 360 nm and emission wavelength of 440 nm.

### Caspase 3 activity

Caspase 3 activity in whole cell extracts was determined spectrophotometrically using the CaspACE™ Assay System, Colorimetric kit (Product code G7220) from Promega (Madison, WI, USA). The method was based on the cleavage of Ac-DEVD-pNA by caspase 3 (DEVDase) to produce a yellow-colored p-nitroaniline (pNA). The pNA produced was monitored spectrophotometrically at 405 nm, as it is a measure of caspase 3 activity. Cells seeded in 6-well plates were treated with 5 and 10 μM CdCl_2_ for 24 h and replicate cells were treated at the same time with an inhibitor of apoptosis, Z-VAD-FMK (50 μM final concentration). In the HO-1 inhibition study, cells were pre-treated with 10 μM SnPPIX for 24 hr before exposure to 5 and 10 μM CdCl_2_ for 24 hr and untreated cells were exposed to medium containing 0.1 % 0.1 M NaOH. After the incubation period, the cells were harvested and caspase 3 activities were determined as described in the kit protocol.

### Intracellular ROS measurement

ROS level was determined by using dihydrofluorescein diacetate (H_2_FDA) method as previously described [37] with little modifications using 50 μM H_2_FDA instead of 20 μM as used in the original method. Cells were incubated with 50 μM H_2_FDA for 30 min and washed with PBS before exposure to 5 and 10 μM CdCl_2_ for 1 h. In the pre-treated experiments, HepG2 cells were incubated with either 10 μM CoPPIX or 10 μM SnPPIX or medium containing 0.1 % 0.1 M NaOH for 24 h, washed with PBS and then treated with 50 μM H_2_FDA for 30 min before 1 h exposure to 5 and 10 μM CdCl_2_. Fluorescence intensity was measured with a fluorescence microplate reader (FL6000) at excitation of 488 nm and emission of 512 nm.

### Mitochondrial and Cytosolic fractions preparation

HepG2 cells were plated in EasyFlask 75 cm^2^ Vent/Close tissue culture flasks (Fisher Scientific, UK) and were allowed to grow to 90 % confluency. The cells were then treated with 5 and 10 μM CdCl_2_ for 24 h. In the induction and inhibition studies, the cells were pre-treated as described above prior to CdCl_2_ exposure. The mitochondrial and cytosolic fractions were prepared as described by Cook et al. [38]. Cell homogenates were prepared in homogenizing buffer as described by Lawal and Ellis [8]. Briefly, after the incubation, the cells were washed with ice-cold PBS and harvested by scrapping with a rubber policeman in PBS. The harvested cells were then centrifuged at 1000 *x g* for 3 min at 4 °C and the cell pellets were resuspended in cold homogenizing buffer (20 mM HEPES-KOH, pH 7.5; 10 mM Sucrose; 10 mM KCl; 1.5 mM MgCl_2_; 1 mM EDTA; 1 mM EGTA; 1 mM DTT; 1 mM PMSF; 2 mg/ml Aprotinin; 10 mg/ml Leupetin; 5 mg/ml Pepstatin). Mitochondrial fractions were prepared by centrifugation of the homogenate at 23,100 *x g* for 30 min at 4 °C. The pellet containing the mitochondrial fractions was resuspended in lysis buffer (50 mM Tris–HCl, pH 7.4; 150 mM NaCl_2_; 0.5 % (v/v) Triton-X-100; 20 mM EGTA; 1 mM DTT; 1 mM Sodium Orthovanadate) and stored at −70 °C. The supernatant was further centrifuged at 100,000 *x g* for 1 hour at 4 °C and the supernatant was retained as cytosolic fractions.

### Western blot analysis

The mitochondrial, cytosolic and whole cell fractions were used for western blot analysis after CdCl_2_ exposure. Approximately 20 μg of total protein was separated by 10 % polyacrylamide gel electrophoresis (SDS-PAGE) [39] and then transferred to nitrocellulose membrane (Hybond, ECL). BCL-xl, HO-1, Caspase 3, Bax, and Cytochrome c protein expressions were detected using anti-BCL-xl, anti-HO-1, anti-Caspase 3, anti-Bax, and anti-Cytochrome c antibodies (1:2000) and a secondary antibody (goat anti-rabbit IG-horseradish peroxidase conjugate) according to the manufacturer’s protocol. Tom40 and GAPDH proteins were detected by Tom40 and GAPDH antibodies (1:2000), respectively, with a goat anti-rabbit antibody. Tom40 and GAPDH were used for the normalization of mitochondria and cytosolic protein loading, respectively, and blots were developed with the ECL luminal chemiluminescence solutions (Cat # RPN 2232) according to the manufacturer’s protocol (GE Healthcare, Buckinghamshire, UK). Protein expressions were detected with an Image reader LAS 3000 and quantitated using ImageJ Software (http://rsb.info.nih.gov/ij/).

### Cell viability assay

The MTT assay method [40] was used to assess cell viability. HepG2 cells were treated in a 96-well plate with 5 and 10 μM CdCl_2_ for 24 h at a concentration of 10^6^ cells/ml in a total volume of 100 μl/well. At the end of the incubation period, 20 μl of MTT (1.2 mg/ml) was added and the cells were allowed to incubate for 4 hours. After the incubation period, the media were discarded and 100 μl of DMSO was added, followed by gently shaking for 10 min to obtain a complete dissolution. Absorbance was read at 560 nm using the Labsystems iEMS microplate spectrophotometer (Vienna, USA). For the caspase 3 inhibition study, HepG2 cells were pre-treated for 1 h with 50 μM Z-DEVD-FMK prepared in cell culture medium (from the 10 mM Z-DEVD-FMK stock prepared in DMSO) prior to CdCl_2_ exposure. After the incubation, the medium was discarded and the cells were washed with PBS before incubating with 5 and 10 μM CdCl_2_ for 24 h. For the HO-1 induction and inhibition studies, HepG2 cells were pre-treated with either 10 μM CoPPIX or 10 μM SnPPIX for 24 h prior to CdCl_2_ exposure. After the 24 h incubation, the medium was discarded and the cells were washed with PBS before incubating with 5 and 10 μM CdCl_2_ for 24 h. Cell viability was determined by MTT assay as described above.

### Apoptosis and necrosis studies

The studies of apoptotic and necrotic cell deaths were carried out using Annexin V-Cy3 Apoptosis Detection Kit Plus from BioVision (Mountain View, CA, USA). Briefly, HepG2 cells were treated with 5 and 10 μM CdCl_2_ for 24 h. In the induction and inhibition studies, the cells were pre-treated with either 50 μM Z-DEV-FMK for 1 h or 10 μM CoPPIX for 24 h prior to CdCl_2_ exposure. After the incubations, the cells were detached using a low trypsin concentration (0.1 %) for one minute. The media containing the cells were centrifuged for 5 min at 1000 *x g*. The cell pellets were then resuspended in 500 μl binding buffer and 5 μl of annexing- V-Cy3 dye and 1 μl of SYTOX Green dye was added to the resuspended cells and mixed thoroughly. The populations of live, apoptotic and necrotic cells were measured using flow cytometry (EPICS XL Coulter; Beck- man, Indianapolis, IN, USA) according to the manufacturer protocol.

### Statistical analysis

Results were analyzed using one way analysis of variance (ANOVA) with Dunnett’s multiple-comparison post-test for comparisons between groups. Statistical analyses were carried out using the Graphpad5 Prism Software. Data were expressed as mean ± SD of three different experiments done in triplicate. Differences were considered statistically significant at p < 0.05.

## Results

### Cd increased calpain and caspase 3 activities, but decreased BCL-xl expression

**Fig. 1:**
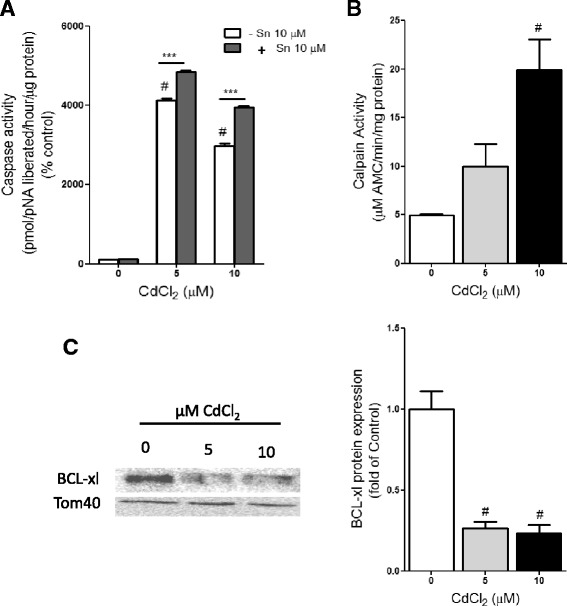
Effects of Cd on pro- and anti- apoptotic proteins in HepG2 cells. HepG2 cells were exposed to 5 and 10 μM CdCl_2_ for 24 h and both whole cell extracts and mitochondrial fraction were prepared from the harvested cells as described in Materials and methods. (A) Caspase 3 activity in the presence and absence of 10 μM SnPPIX and (B) Calpain activities was determined in the whole cell extracts. Values represent mean ± SD for 3 separate experiments (n = 3). (C) The expression of mitochondrial BCL-xl protein was analyzed by western blot using antibodies to BCL-xl or Tom40 as internal control for protein loading. Quantification of protein expressions was done with image j. Data represent the mean ± SD of three different experiments (n = 3). ^#^p < 0.0001 as significant differences between Cd-treated and control. ***p < 0.0001 as Significant differences between SnPPIX pre-treated and non pre-treated cells

Different studies using different models have shown the involvement of caspase 3 in Cd-induced apoptosis [13, 15, 22]. In the present study, we investigated whether Cd can activate the caspase 3-dependent pathway under our experimental conditions using HepG2 cells exposed to 5 and 10 μM CdCl_2_ for 24 h. The data showed that Cd caused significant increases in caspase 3 activity in HepG2 cells (Fig.1A), being 41.3- and 29.8-fold at 5 and 10 μM CdCl_2_, respectively. Our results also showed that the presence of SnPPIX significantly enhanced the increase in caspase 3 activity induced by 5 and 10 μM CdCl_2_. Also, several studies have indicated that calpain can activate caspase 3 directly, thereby providing a link between calpain and caspase 3-dependent apoptosis. Therefore, to examine whether calpain activation is involved in Cd-induced cell death in HepG2 cells, we evaluated calpain activity in the cells after CdCl_2_ exposure. The results showed a nonsignificant increase in calpain activity in HepG2 cells exposed to 5 μM CdCl_2_ and a significant 4-fold increase at 10 μM CdCl_2_ (Fig.1B). To further confirm the involvement of the caspase 3-dependent apoptotic pathway in HepG2 cells after Cd exposure, we examined the expression levels of mitochondrial BCL-xl, an anti-apoptotic protein, after Cd exposure. The results showed a 7.3 and 7.7- fold decrease in BCL-xl expressions at 5 and 10 μM CdCl_2_, respectively when compared to the control (Fig. 1C). These results showed that the caspase 3-dependent pathway may be involved in Cd-induced apoptosis in HepG2 cells and calpain activation may be involved in Cd-induced cell death at higher concentrations.

### Cd upregulated expression of heme oxygenase-1

**Fig. 2:**
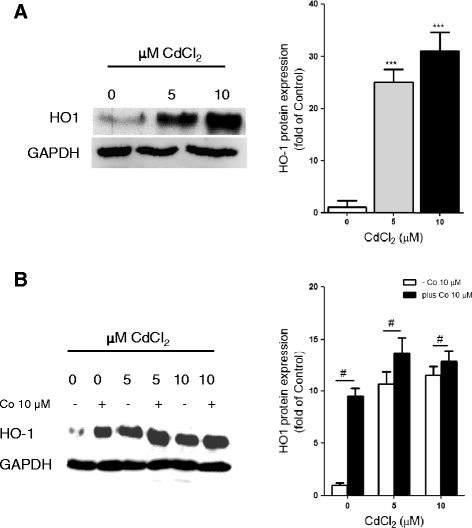
Effects of Cd on heme-oxygenase-1 expression. HepG2 cells were (A) treated with 5 and 10 μM CdCl_2_ for 24 h and (B) pre-treated with 10 μM CoPPIX for 24 h before exposure to 5 and 10 μM CdCl_2_ for 24 h. The expression of HO-1 protein was determined in the whole cell lysates by western blot analysis and GAPDH was used as loading control. Data represent the mean ± SD of three different experiments (n = 3). Significant difference between Cd-treated and control (***p < 0.0001). ^#^p < 0.01 as significant difference between CoPPIX pre-treated and non pre-treated cells

In order to examine the response of the HepG2 antioxidant system to Cd-induced insults, we evaluated the expression of the cytoprotective heme oxygenase-1 (HO-1) enzyme, an inducible isoform of heme oxygenases, after Cd exposure (Fig. 2A). The data showed 25 and 31- fold increases in HO-1 expression after treatment with 5 and 10 μM CdCl_2_, respectively for 24 h (Fig. 2A). This suggested the involvement of free radical generation in the cytotoxicity of Cd.

In order to confirm that CoPPIX, a well known pharmacological inducer of HO-1, has an inducible effect on HO-1 in our study model, we examined the HO-1 expression in HepG2 cells pre-treated with 10 μM CoPPIX for 24 h prior to Cd exposure (Fig. 2B). The data showed that the presence of CoPPIX alone triggered a 10-fold induction of the HO-1 expression from its basal level (Fig. 2B). The presence of CoPPIX also caused significant increases of 27.29 and 11.34 %, in HO-1 expression at 5 and 10 μM CdCl_2_, respectively. The increases in HO-1 expression in the presence of either CoPPIX alone or 5 μM or 10 μM CdCl_2_ is not additive when compared to the CoPPIX pre-treated cells (Fig. 2B). These data showed that the presence of CoPPIX has an inducible effect on HO-1 expression and that CoPPIX acts non-additively with Cd triggering an increased HO-1 expression in the HepG2 cells.

### Heme oxygenase-1 attenuated ROS production

**Fig. 3:**
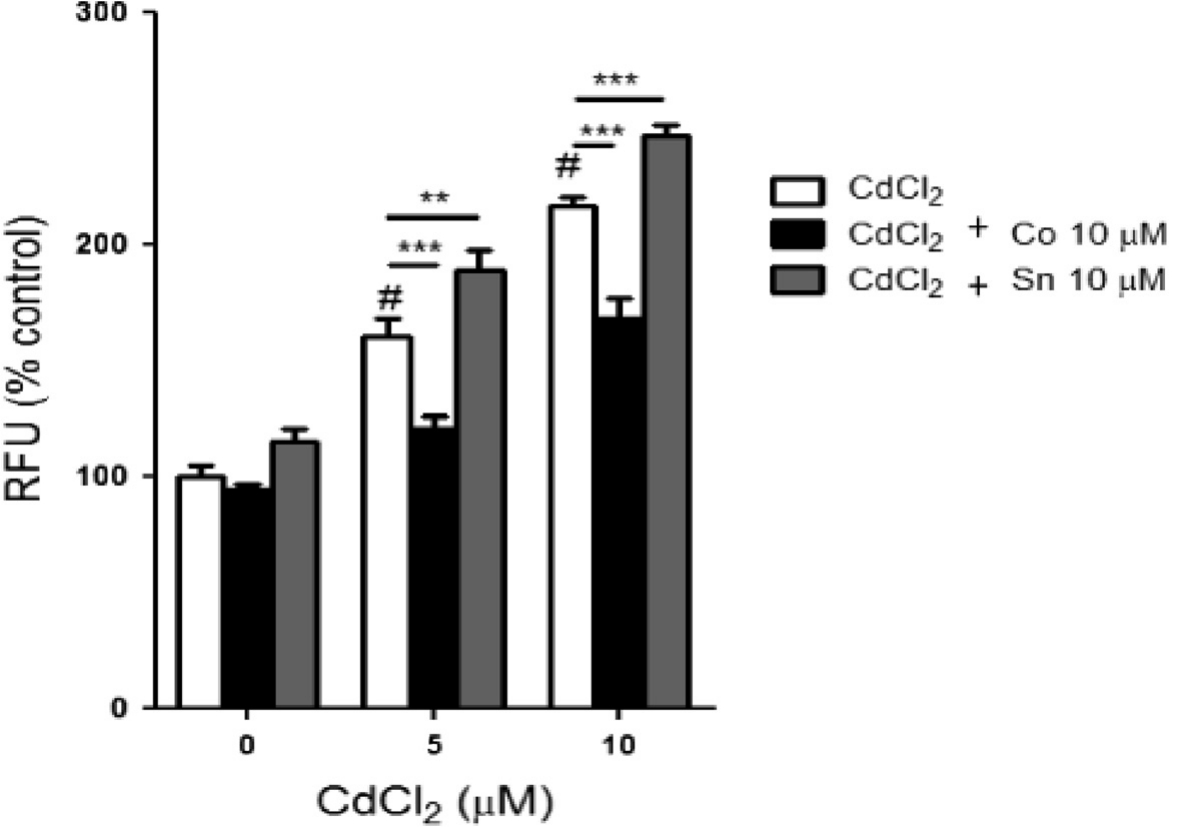
HO-1 attenuates Cd-induced oxidative stress. HepG2 cells were either treated with 5 and 10 μM CdCl_2_ for 1 h or pre-treated with either 10 μM CoPPIX or 10 μM SnPPIX for 24 h before exposure to 5 and 10 μM CdCl_2_ for 1 h. ROS levels were determined by the H_2_FDA method as described in the Materials and methods. Values shown are mean ± SD of three different experiments done in triplicate (n = 3). # p < 0.0001 as significant differences between Cd-treated and control. *** p < 0.001 as a significant difference between pre-treated and non pre-treated cells

In order to define the role of HO-1 in modulating Cd-induced oxidative stress, we exposed HepG2 cells to either 10 μM CoPPIX or 10 μM SnPPIX before treatment with CdCl_2_ and then assessed the levels of ROS production. Our data showed that Cd caused a 60.00 and 116.67 % increase in ROS production at 5 and 10 μM CdCl_2_, respectively (Fig. 3). However, the presence of CoPPIX significantly reduced ROS production by 24.60 and 22.20 % at 5 and 10 μM CdCl_2_, respectively, while SnPPIX caused significant 18.34 and a 13.86 % increase in ROS production at 5 and 10 μM CdCl_2_, respectively (Fig. 3). This data showed that HO-1 attenuates the Cd-induced oxidative stress in HepG2 cells.

### Heme oxygenase-1 attenuated the pro-apoptotic effects

**Fig. 4:**
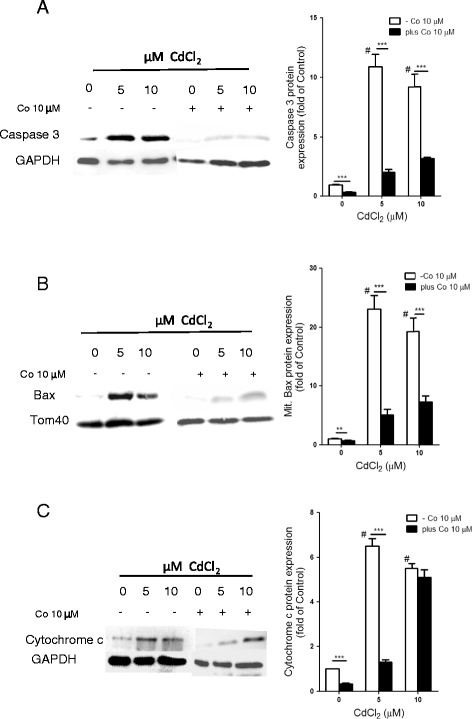
Induction of HO-1 attenuates the pro-apoptotic effects of Cd. HepG2 cells were either exposed to 5 and 10 μM CdCl_2_ for 24 h or pre-treated with 10 μM CoPPIX for 24 h before treated with 5 and 10 μM CdCl_2_ for 24 as described in the Materials and methods. (A) Whole cell extracts caspase 3 protein (B) Mitochondrial Bax protein and (C) Cytosolic cytochrome c protein expressions were analyzed by western blot. Values are mean ± SD of three different experiments done in triplicate (n = 3). ^#^p < 0.0001 as significant differences between Cd-treated and control. ***p < 0.0001, **p < 0.001 as significant differences between CoPPIX pre-treated and non pre-treated cells

Heme oxygenase-1 has been shown in several studies to protect against oxidative stress and oxidative damage in different cell lines [41–44]. Increased ROS and other free radicals have been implicated in apoptosis and necrosis [26, 27, 45]. In order to define the role of HO-1 in Cd-induced apoptosis and necrosis in HepG2 cells, we induced HO-1 expression using a well known HO-1 inducer, CoPPIX. HepG2 cells were pre-treated with 10 μM CoPPIX for 24 h before treatment with 5 and 10 μM CdCl_2_ for 24 h and the protein expression of caspase 3, mitochondrial Bax, and cytosolic cytochrome c were examined by western blot (Fig. 4). The results showed that Cd caused significant increases in the expression of caspase 3 (11 and 9- fold), mitochondrial Bax (23 and 19- fold) and cytochrome c (7 and 6-fold) in HepG2 cells after exposure to 5 and 10 μM CdCl_2_, respectively (Fig. 4). The presence of HO-1-induction, however attenuated the Cd-induced expression of these pro-apoptotic markers. HO-1 caused a 5 and 3- fold reduction in caspase 3 expression (Fig. 4A); a 4.5 and 2.6- fold reduction in mitochondrial Bax expression (Fig. 4B) and a 5-fold decrease in cytosolic cytochrome c expression at 5 μM CdCl_2_, respectively (Fig. 4C). This data suggest the cytoprotective effect of HO-1 in attenuating Cd-induced caspase 3-dependent pathway of apoptosis.

### Heme oxygenase-1 attenuated Cd-induced cytotoxicity

**Fig. 5:**
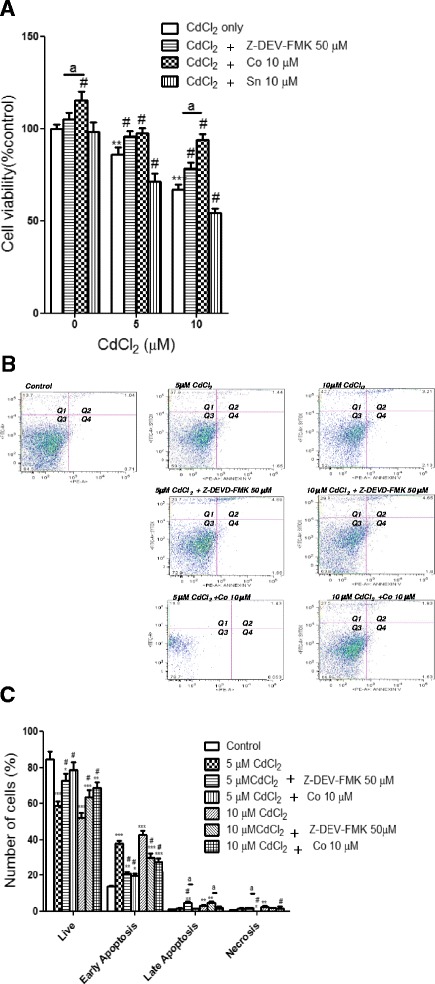
Effects of HO-1 and caspase 3 on Cd-induced apoptosis and necrosis in HepG2 cells. HepG2 cells were either exposed to 5 and 10 μM CdCl_2_ for 24 h, or 50 μM Z-DEVD-FMK for 1 h before exposure to 5 and 10 μM CdCl_2_ for 24 h, or either 10 μM CoPPIX or 10 μM SnPPIX for 24 h before exposure to 5 and 10 μM CdCl_2_ for 24 h. (A) Cell viability was determined by MTT assay. Data represent the mean ± SD of three different experiments done in triplicate (n = 3). HepG2 cells were harvested and stained with annexin V-Cy3 and SYTOX dyes. (B) The stained cells were examined under the flow cytometry using annexin-V/PE-A and the SYTOX/FITC channels. (C) Histogram representative of the population of cells (10,000 events) distribution in Q1, early apoptosis; Q2, late apoptosis; Q3, live; Q4, necrosis. *p < 0.05, **p < 0.001, ***p < 0.0001 as significant differences between Cd-treated and control. ^#^p < 0.0001 as a significant difference between pre-treated cells and cells exposed to Cd alone. ^a^p < 0.01 as significant differences between a Z-DEVD-FMK pre-treated and CoPPIX pre-treated cells

In order to evaluate whether HO-1 induction would attenuate Cd-induced cytotoxicity and to further demonstrate the involvement of caspase 3 in Cd cytotoxicity in HepG2 cells, the cells were pre-treated with either 10 μM CoPPIX (HO-1 inducer) or 10 μM SnPPIX (HO-1 inhibitor) for 24 h or 50 μM Z-DEVD-FMK (caspase 3 inhibitor) for 1 h, whereafter the cells were further exposed to 5 and 10 μM CdCl_2_ for 24 hours. Cell viability was determined using the MTT assay. The results showed that Cd significantly decreased cell survival at 5 (p < 0.05) and 10 (p < 0.001) μM. Cd caused a 13.89 and 32.53 % decrease in cell survival at 5 and 10 μM, respectively (Fig. 5A). The presence of Z-DEVD-FMK attenuated the cytotoxic effects of Cd with a significant (p < 0.01) increase in cell survival at 5 μM CdCl_2_ when compared with CdCl_2_-only treated HepG2 cells. Similarly, CoPPIX pre-treated cells showed significantly increased in cell survival by 11.61 and 26.97 % at 5 and 10 μM CdCl_2_, respectively when compared to cells exposed to Cd only (Fig. 5A). HO-1 induction also caused a 15.67 % increase in cell survival at the basal level. In addition, the presence of CoPPIX caused a significant increase both in the basal level (10.33 %) and at 10 μM CdCl_2_ (15.67 %) when compared to the presence of Z-DEVD-FMK (Fig. 5A). However, the increased cell viability caused by CoPPIX at 5 μM was not significant when compared to Z-DEVD-FMK. This suggested that caspase 3 activation via ROS production may be the predominant pathway responsible for Cd-induced cell death at 5 μM CdCl_2_ since both CoPPIX and Z-DEVD-FMK produced similar effects at this concentration. However, the presence of SnPPIX caused a decrease in cell survival compared to cells exposed to Cd only. These results suggest that HO-1 induction and/or casapse 3 inhibition may be important in attenuating Cd-induced cytotoxicity.

### Heme oxygenase-1 attenuated Cd-induced apoptosis and necrotic cell death

To further demonstrate the cytoprotective effect of HO-1 in the Cd-induced caspase 3-dependent apoptotic pathway in HepG2 cells, the cells were pre-treated with either 10 μM CoPPIX for 24 h or 50 μM Z-DEVD-FMK for 1 h before exposure to 5 and 10 μM CdCl_2_ for 24 h. After treatments, cells were harvested, stained with annexin v-cy 3 and SYTOX dyes and examined using flow cytometry, while cell population distribution was quantified also using flow cytometry (Fig. 5B & C). The results showed that 5 μM CdCl_2_ caused a 39.34 % and 1.65 % apoptotic and necrotic cell death, respectively, while 45.91 % and 2.13 % of cells exposed to 10 μM were apoptotic and necrotic cells, respectively (Fig. 5B & C). Both 5 and 10 μM CdCl_2_ caused significant reductions in the population of the live cells (Fig. 5B & C). The presence of CoPPIX significantly increased the population of the live cells by 19.70 % and 16.90 % at 5 and 10 μM CdCl_2_, respectively. Z-DEVD-FMK also caused significant increases of 13.90 % and 11.80 % at 5 and 10 μM CdCl_2_, respectively. In contrast, COPPIX and Z-DEVD-FMK both significantly reduced the number of early apoptotic cells at 5 and 10 μM CdCl_2_ (Fig.5B & C). However, the population of late apoptotic cells in CoPPIX pre-treated cells was significantly lower than that of Z-DEVD-FMK at both 5 and 10 μM CdCl_2_. Similarly, COPPIX pre-treatment significantly reduced the population of necrotic cells by 1.59 % and 0.50 % at 5 and 10 μM CdCl_2_, respectively. On the other hand, Z-DEVD-FMK did not cause a significant reduction in the necrotic cell population at both of these concentrations. The population of the necrotic cells in COPPIX pre-treated HepG2 cells was significantly lower than that in Z-DEVD-FMK pre-treated cells exposed to 5 μM CdCl_2_.

## Discussion

Our data indicate that Cd upregulated HO-1 expression, which modulated the antioxidant and anti-apoptotic effects, thus limiting the cytotoxic, prooxidative and pro-apoptotic effects of Cd, especially when HO-1 was induced prior to Cd exposure.

We hypothesize that HO-1 upregulation, prior to Cd exposure, can serve as a protective mechanism against Cd-induced oxidative stress and cytotoxicity. Our data indicated that the induction of HO-1 significantly increased cell survival most probably as a result of decreased oxidative stress. The data also indicated that the cell death induced by Cd involved the mitochondrial-caspase 3 dependent apoptosis pathway, which may or may not involve calpain activation. In addition, this study showed that Cd induced largely apoptotic cell death at the lower dose of 5 μM and both apoptotic and necrotic cell death at the higher dose of 10 μM in confirmation with our earlier study done in HEK 293 cells exposed to the same doses of Cd [10].

**Fig. 6:**
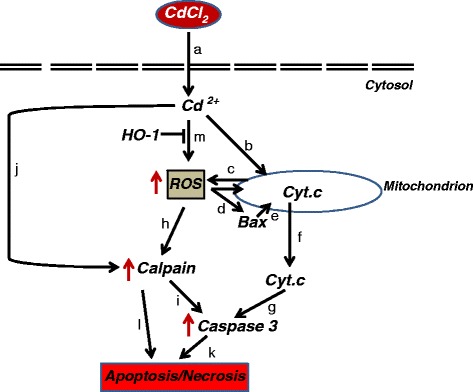
Proposed mechanisms for HO-1 intervention in Cd-induced apoptosis and necrosis in HepG2 cells. CdCl_2_ dissociates in the cells to produce Cd ion (Cd^2+^) (a), which can induce mitochondrial permeability transition pore by modifying the thiol groups of the membrane proteins (b), resulting in the leakage of electrons to generate ROS (c). ROS can activate Bax (d) and resulting in its migration into the mitochondrial membrane (e) with the consequent release of cytochrome c into the cytosol (f). The cytochrome c caused the activation of caspase 3 to initiate apoptosis (g). ROS generated could also activate calpain (h) which can further activate caspase 3 (i). Cd^2+^ can also directly activate calpain by mimicking Ca^2+^ ion (j). The activation of caspase 3 and calpain can result in apoptosis (k) and necrosis (l) respectively. HO-1 can attenuate ROS produced either by direct effects of Cd^2+^ on cellular molecules (m) or by indirect effects of Cd^2+^ on mitochondrial membrane permeability (c), resulting in calpain inhibition and blockage of Bax mobilization to the mitochondrial membrane

The present study also confirms earlier reports that have implicated Cd in the induction of apoptosis and necrotic cell death in HepG2 cells [23, 46, 47]. The use of Z-DEVD-FMK as an effective inhibitor of caspase 3 in HepG2 cells has already been well reported in different studies [48, 49]. In this present study, Z-DEVD-FMK prevented Cd-induced cell death at both 5 and 10 μM CdCl_2_, indicating the involvement of caspase 3 in cell death at both of these concentrations. The activation of caspase 3 may be due to the direct effect of cadmium ion (Cd^2+^) on Bax protein with the latter inducing mitochondrial membrane permeability transition with the consequent release of cytochrome c into the cytosol (Fig. 6). These present findings correlate with the earlier work done by Oh and Lim [46]. They found that Cd-induced apoptosis in HepG2 cells in a time- and dose- dependent manner and this induction correlated with mitochondrial Bax cleavage and cytosolic cytochrome c release. BCL-xl, an anti-apoptotic protein, prevents the efflux of cytochrome c from the mitochondria into the cytosol thereby inhibiting apoptosis [50]. Our study revealed that Cd significantly depletes the level of mitochondrial BCL-xl protein and that Cd caused a correlated increase in both caspase 3 activity and expression in HepG2 cells at 5 and 10 μM, which may partly be due to an increased in ROS and calpain activity. However, the lower activity and expression seen at 10 μM CdCl_2_ may be due to the inhibitory effects of Cd at this concentration. Cd has been shown in a previous study to inhibit caspase 3 activity with an IC50 of 9 μM [51]. The lower caspase 3 activity observed at 10 μM may be due to the displacement of Zn from its caspase 3 binding sites. In addition, Cd may interfere (in a concentration dependent manner) with the activation or function of upstream caspases, such as caspase −8 or −9, leading to the reduction in the expression and amount of active caspase 3.

HO-1 is a cytoprotective enzyme and its expression is under the control of the Nrf2 transcription factor. The presence of oxidative stress triggers the release of Nrf2 from its keap1 repressor in the cytosol and its consequent migration into the nucleus where it heterodimerises with small Maf proteins [52] to stimulate the transcription of downstream genes, such as HO-1, through its binding to the antioxidant response element (ARE) at the promoter end of the responsive gene [53]. It has already been reported that HO-1 prevented crontonaldehyde-induced apoptosis in HepG2 cells [34]. The use of CoPPIX, a known inducer of HO-1 [42, 54], in this study, caused significantly decreased levels of mitochondrial Bax, cytosolic cytochrome c and caspase 3 protein expression, implicating HO-1 upregulation in the modulation of Cd-induced apoptosis. However, HO-1 induction did not cause a significant reduction in cytochrome c expression at 10 μM CdCl_2_ (Figure 4C). HO-1 is not a scavenger of Cd^2+^ but acts through its byproducts to scavenge ROS. It is therefore possible that Cd^2+^ may contribute significantly to the release of cytochrome c through its direct effect on mitochondrial membrane permeability transition pore, especially at higher doses (10 μM CdCl_2_), such that modulation by HO-1 induction may not have significant effects on the cytosolic cytochrome c level at this dose of CdCl_2_. Indeed, the modulation of HO-1 expression significantly increased cell survival and also caused a significant reduction in the population of apoptotic and necrotic cells after CdCl_2_ exposure.

Oxidative stress, defined as an imbalance between the level of ROS and the antioxidant defence system with the former being favoured, if not managed, can lead to oxidative damage with its associated consequences of damage to important cellular macromolecules like DNA, proteins and lipids. HO-1 has been shown to protect HepG2 from H_2_O_2_-induced oxidative stress [41]. The data from the present study also highlight the involvement of oxidative stress in mediating Cd-induced apoptosis and necrosis in HepG2 cells as seen in the high level of ROS produced after Cd exposure. The upregulation of HO-1, however, attenuated ROS production with a corresponding reduction in the levels of Bax, cytochrome c and caspase 3 levels.

Calpain belongs to the family of Ca^2+^-dependent cysteine proteases [55]. The involvement of calpain in apoptosis and its activation by Ca^2+^ is well established [55]. Calpain can activate caspase 3 thereby providing a link with the mitochondrial caspase 3 dependent pathways in apoptosis [16, 56]. Cd^2+^, with the same ionic radii as Ca^2+^, mimics Ca^2+^ in the cells and thus can activate calpain (Fig. 6). Indeed, it has been shown that Cd activates calpain in the kidney proximal tubule cells [14], and in human embryonic kidney (HEK 293) cells [10]. In the present study, we have shown that Cd caused significant activation of calpain at 10 μM. We have previously shown that Cd caused a significant increase in calpain activity in HEK 293 cells at 10 μM [10]. The increased calpain activity may be due to the direct effects of Cd^2+^ on calpain (Fig. 6) or may be mediated by increased intracellular Ca^2+^ [10] or by increased ROS production [57]. The significant elevation in caspase 3 activities at 5 μM without a significant increase in calpain activity showed that calpain activation may not be required for casapse 3 activation at this dose. However, both calpain and caspase 3 were significantly activated at 10 μM, indicating the involvement of calpain in caspase 3 activations and this may account for the high necrotic cell population at this dose. HO-1 upregulation, however, attenuated but did not completely eliminate the population of necrotic cells, probably by reducing the ROS generation induced by Cd.

## Conclusion

The present study demonstrates that HO-1 protected against Cd-induced caspase 3 apoptosis by attenuating oxidative stress and therefore could serve as a potential therapeutic target to prevent the hepatotoxic effects of Cd.

## Abbreviations

CoPPIX: Cobalt Protoporphrin IX
Z-DEVD-FMK: Z-Asp-Glu-Val-Asp-fluoromethylketone
